# The Anti-Tumorigenic Role of Cannabinoid Receptor 2 in Non-Melanoma Skin Cancer

**DOI:** 10.3390/ijms24097773

**Published:** 2023-04-24

**Authors:** Jennifer Ana Iden, Bitya Raphael-Mizrahi, Aaron Naim, Albert Kolomansky, Tamar Liron, Drorit Neumann, Marilena Vered, Yankel Gabet

**Affiliations:** 1 Department of Anatomy and Anthropology, Sackler Faculty of Medicine, Tel Aviv University, Tel Aviv 69978, Israel; jenniferiden@mail.tau.ac.il (J.A.I.);; 2 Department of Cell and Developmental Biology, Sackler Faculty of Medicine, Tel Aviv University, Tel Aviv 69978, Israel; 3 Department of Oral Pathology, Oral Medicine and Maxillofacial Imaging, The Goldschleger School of Dental Medicine, Sackler Faculty of Medicine, Tel Aviv University, Tel Aviv 69978, Israel; 4 Institute of Pathology, The Chaim Sheba Medical Center, Tel Hashomer, Ramat Gan 52621, Israel

**Keywords:** non-melanoma skin cancer, cannabinoid receptor 2, tumor regression, tumor microenvironment, myeloid-derived suppressor cells, T cells

## Abstract

Five million non-melanoma skin cancers occur globally each year, and it is one of the most common malignant cancers. The dysregulation of the endocannabinoid system, particularly cannabinoid receptor 2 (CB2), is implicated in skin cancer development, progression, and metastasis. Comparing wildtype (WT) to systemic CB2 knockout (CB2^-/-^) mice, we performed a spontaneous cancer study in one-year old mice, and subsequently used the multi-stage chemical carcinogenesis model, wherein cancer is initiated by 7,12-dimethylbenz[a]anthracene (DMBA) and promoted by 12-O-tetradecanoylphorbol-13-acetate (TPA). We found that aging CB2^-/-^ mice have an increased incidence of spontaneous cancerous and precancerous skin lesions compared to their WT counterparts. In the DMBA/TPA model, CB2^-/-^ developed more and larger papillomas, had decreased spontaneous regression of papillomas, and displayed an altered systemic immune profile, including upregulated CD4+ T cells and dendritic cells, compared to WT mice. Immune cell infiltration in the tumor microenvironment was generally low for both genotypes, although a trend of higher myeloid-derived suppressor cells was observed in the CB2^-/-^ mice. CB2 expression in carcinogen-exposed skin was significantly higher compared to naïve skin in WT mice, suggesting a role of CB2 on keratinocytes. Taken together, our data show that endogenous CB2 activation plays an anti-tumorigenic role in non-melanoma skin carcinogenesis, potentially via an immune-mediated response involving the alteration of T cells and myeloid cells coupled with the modulation of keratinocyte activity.

## 1. Introduction

Non-melanoma skin cancer consists of two main types: squamous cell carcinoma (SCC) and basal cell carcinoma (BCC). Around the world, BCC accounts for around 80% and SCC accounts for about 20% of all diagnosed NMSC cases [1,2,3,4]. While metastasis in cases of BCC is rare, metastasis of SCC is more common and potentially fatal, wherein 20% of all skin cancer related deaths are caused by metastatic SCC [5]. Incidences of NMSC are increasing annually, particularly in the aging Caucasian population, and NMSC is the most frequent acquired cancer and one of the most common malignant cancers [1,3,4].

To investigate the role of cannabinoid receptor 2 (CB2) in skin carcinogenesis, we used the multi-stage chemical carcinogenesis model, wherein a tumor initiator (7,12- dimethylbenz[a]anthracene, DMBA) is applied topically, followed by a tumor promoter (12-O-tetradecanoylphorbol-13-acetate, TPA) [6]. This model is well-established, known to mimic the stages of SCC in humans, and has been used to demonstrate that inflammation is a key process in skin tumorigenesis, being regulated by NF-κB, and involving the pro-inflammatory cytokines TNFα, IL-17, and IL-6 [7,8,9]. This model induces an accumulation of mutations, specifically in Ras family proteins (*Kras*, *Hras1*, and *Nras*) and *p53*, which occur at high frequency in human NMSC [10]. Importantly, the DMBA/TPA model has shown the role of thymic stromal lymphoprotein (TSLP) in tumor development, wherein TSLP, produced predominately by epithelial cells, activates myeloid DCs which trigger the production of Th2 cells [11,12]. Multiple studies have established that TSLP exhibits anti-tumor activity in keratinocytes [13,14]. In addition to TSLP, the functional phenotypes of T cells are also dependent on GATA-3 [15,16] and in instances of SCC progression, it is significantly down-regulated [17]. It has also been shown that an IL-17 mediated decrease in pro-tumor myeloid-derived suppressor cells (MDSC) can suppress skin carcinogenesis [18]. Similarly, in humans and mice, upregulated polymorphonuclear myeloid-derived suppressor cells (PMN-MDSC) and monocytic myeloid-derived suppressor cells (M-MDSC) are associated with a poor prognosis and a strong immunosuppressive effect leading to tumor progression [19].

The role of immunosuppression is observed clinically in patients receiving organ transplants; in these immunosuppressed patients, incidences of squamous cell carcinoma are higher, coupled with decreased rates of spontaneous regression [20,21]. In addition to the immunological basis for spontaneous regression, non-immunological mechanisms have been shown, including the endocannabinoid system [22,23,24].

Targeting the endocannabinoid system, and in particular cannabinoid receptor 2 (CB2), has been repeatedly proposed as a promising option against a vast array of cancer types, ranging from hematological to solid tumors [25,26,27,28,29]. This was mainly based on the observation that CB2 expression increases in several cancerous tissues, and NMSC cells and papilloma cells express CB2 receptors [30]. Endogenous CB2 activation can interfere with endothelial cell migration, inhibition of growth, impaired vascularization, and apoptosis of tumor cells [31]. However, CB2 has been shown to have seemingly opposing effects in skin cancer development depending on the model, i.e., activation of the receptor leads to tumor cell death or promotes tumor growth [32,33].

Recently, we reported on the protective role of CB2 in colon cancer and found that colon cancer incidence in humans is significantly associated with polymorphism in the CNR2 gene encoding for CB2 [34]. In mice lacking CB2, the severity of chemically- and genetically-induced colon cancer was higher than in wild-type mice. Cancer severity was likely associated with an unfavorable immuno-suppressive environment, suggesting that the protective role of CB2 may extend to other types of cancer. Here, we focus on the role of CB2 in potentiating both the immunological and non-immunological response against tumor development in the skin.

## 2. Results

### 2.1. CB2^-/-^ Is Associated with an Increased Risk for Spontaneous Skin Cancer in Males

**Figure 1:**
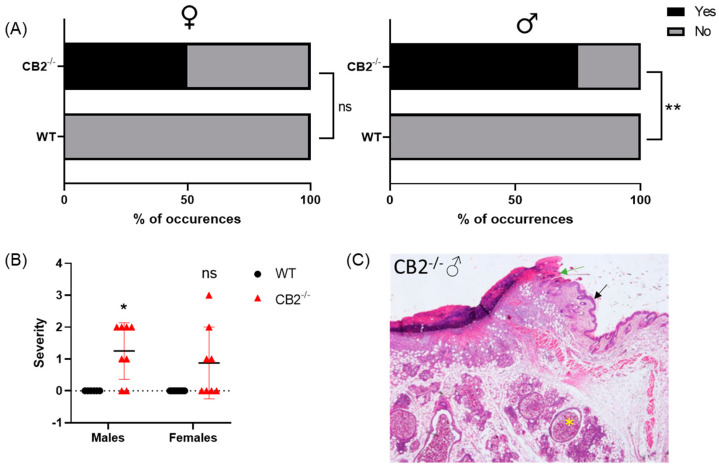
CB2^-/-^ is associated with increased risk of skin cancer in aged male mice. (**A**) Odd’s ratio in CB2^-/-^ mice to develop cancerous and pre-cancerous lesions (“yes” occurrences) in the skin as assessed histopathologically in females (left) and males (right); ** *p* < 0.01, ns: not significant versus WT mice for females, Fisher’s exact test. (**B**) Severity of dysplasia according to the scale: 0–normal tissue; 1–chronic inflammation, ulceration, atrophy, or hyperplasia; 2–cellular atypia, metaplasia, or mitotic figures; 3–dysplasia; 4–positive carcinoma. (**C**) Histological section of left flank of CB2^-/-^ male stained with hematoxylin and eosin; epidermis (black arrow), ulceration of the skin (green arrow), and dermal cystic structures (yellow star). Original magnification ×40. Mann–Whitney *U* test, * *p* < 0.05.

WT mice grown in SPF conditions until the age of one year do not typically develop cancer. To examine whether CB2 contributes to the natural resistance to spontaneous cancer, we performed a histopathological screen of 14-month-old WT and CB2^-/-^ male and female mice. A careful analysis of the tissues collected from the skin revealed the occurrence of cancerous and pre-cancerous lesions in CB2^-/-^ mice in a sex-preferential manner. CB2^-/-^ male mice demonstrated a significant increase in cancerous and precancerous lesions incidence (*p* = 0.010, Figure 1A) and severity (*p* = 0.015, Figure 1B,C). In CB2^-/-^ female mice, there was an increased occurrence (50%) of pre-cancerous lesions in the skin, but this difference was not statistically significant.

### 2.2. CB2^-/-^ and WT Mice Have Similar Lymphoid Compartments in the Spleen and Bone Marrow at Baseline

**Figure 2:**
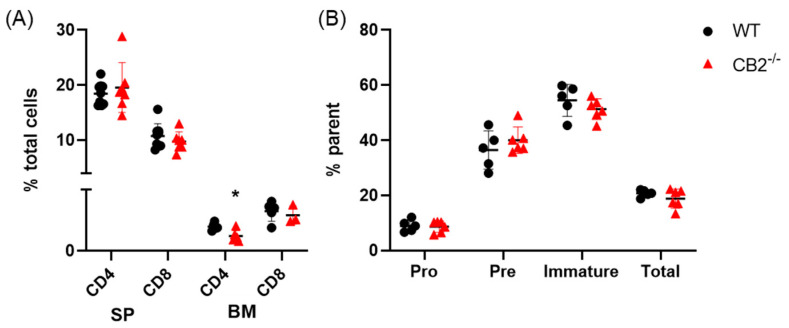
CB2^-/-^ male mice exhibit similar lymphoid cell populations in the spleen and bone marrow. (**A**) Relative frequency of CD4+ and CD8+ T cells in the spleen (SP) and bone marrow (BM). (**B**) Relative frequency of pro-B cells (B220^+^ IgM^−^ CD43^high^) and pre-B cells (B220+ IgM^−^ CD43^low^), immature (B220+ IgM+) and total B cells (B220+) in the bone marrow. All values expressed as percent of total cells or parent population as indicated. WT, n ≥ 4; CB2^-/-^, n ≥ 4. Student’s *t* test, * *p* < 0.05.

Previous reports have shown that CB2^-/-^ males at baseline exhibit elevated neutrophils and monocytes in the bone marrow, yet the number of these cells is similar in the spleen and peripheral blood compared to their WT counterparts [35]. To the best of our knowledge, there are no reports on differences in CB2^-/-^ mice in the lymphoid compartment in the spleen and bone marrow. Therefore, we assessed the spleen for T cells (CD4+ and CD8+) and the bone marrow for T cell and B cell subpopulations in naïve male mice (12-week-old). We found the T and B cell populations to be similar across both genotypes, both occurring at 20% frequency in the spleen and bone marrow respectively, with a significant 38% decrease in CD4+ T cells (*p* = 0.010) in the bone marrow only of CB2^-/-^ mice (Figure 2). All values were in the normal range reported for C57BL/6J mice [36,37,38].

### 2.3. CB2^-/-^ Mice Have Heightened Tumor Susceptibility in the DMBA/TPA Model

**Figure 3:**
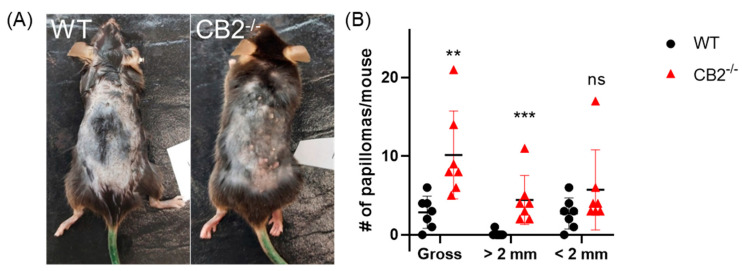
CB2 receptor protects against papilloma development in DMBA/TPA model. (**A**) Representative image of papillomas on the backs of WT and CB2^-/-^ males after 27 weeks of cancer induction. (**B**) Gross number of papillomas, papillomas less than 2 mm and greater than 2 mm. Mann–Whitney U test, ** *p* < 0.01, *** *p* < 0.001, ns, not significant.

Because CB2^-/-^ males showed significantly enhanced spontaneous precancerous lesions, DMBA/TPA treatment was performed in males. In WT and CB2^-/-^ mice receiving DMBA/TPA treatment, papillomas began to appear around 17 weeks after cancer induction and were subsequently recorded weekly. An analysis of papilloma incidence over time showed a significant difference between WT and CB2^-/-^ mice wherein the CB2^-/-^ mice had significantly more tumors, despite the fluctuations (Figure 3A,B). Indeed, some lesions subsided spontaneously. After 27 weeks of cancer induction, papillomas were counted and the diameter of each lesion was measured. At this time, there were more lesions in the CB2^-/-^ mice, averaging ten papillomas per mouse, than in the WT controls, averaging less than three papillomas per mouse (*p* = 0.007, Figure 3B). Interestingly, CB2 knockout not only resulted in more tumors, but also affected their size. While no significant difference between groups was found for the occurrence of small papillomas (less than 2 mm), CB2^-/-^ mice had significantly more papillomas with diameters greater than 2 mm (*p* = 0.003, Figure 3B). Indeed, WT mice had virtually no large tumors whereas all the CB2^-/-^ mice had between 2 and 11 large lesions.

### 2.4. CB2^-/-^ Mice Have Depleted Spontaneous Regression of Papillomas in the DMBA/TPA Model

**Figure 4:**
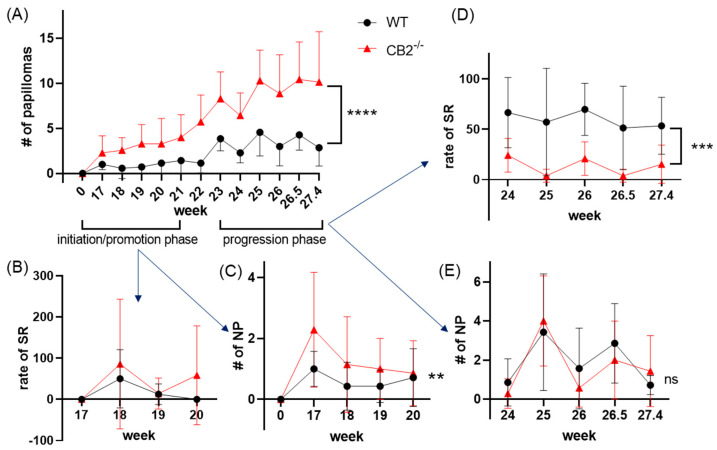
CB2^-/-^ mice have differential papilloma formation and spontaneous regression in the initiation/promotion and progression phase of the DMBA/TPA model. (**A**) Gross number of papillomas, n = 7 per group, 2− way ANOVA for repeated measures, **** *p* < 0.0001. (**B**) Rate of papillomas in spontaneous regression (percent papillomas regressed from the previous measurement, SR) during the initiation/promotion phase; no statistical analysis due to many undefined values (0 regression events/0 papillomas), only mice with defined values are included. (**C**) Number of newly formed papillomas (NP) in the initiation/promotion phase, n = 7 per group, 2−way ANOVA, WT vs. CB2^-/-^ only, ** *p* = 0.01. (**D**) Rate of SR in the progression phase, analysis performed from week 24 onward due to many undefined values, as in B. n = 7 per group, 2−way ANOVA WT vs. CB2^-/-^ only, *** *p* = 0.004. (**E**) Number of newly formed papillomas in the progression phase, 2−way ANOVA WT vs. CB2^-/-^ only, ns, not significant.

As mentioned above, CB2^-/-^ mice showed enhanced growth of larger papillomas compared to WT. CB2^-/-^ mice showed significantly higher gross papilloma formation throughout the experiment (*p* < 0.0001, Figure 1A). We observed after 19 weeks (post DMBA application) that many WT mice were experiencing the spontaneous regression of the papillomas. From this time, we mapped the papillomas and recorded percent regression. Interestingly, no differences in the percent of spontaneously regressing papillomas were seen in the initiation/promotion phase (Figure 4B), although new tumor formation was significantly higher in the CB2^-/-^ mice (*p* = 0.01, Figure 4C, Table S1). However, during the later progression phase, we observed no significant differences in new tumor formation (Figure 4E), but enhanced spontaneous regression in WT mice, occurring at twice the rate compared to CB2^-/-^ mice (*p* = 0.004, Figure 4D, Table S1).

### 2.5. CB2^-/-^ and WT Mice Treated with DMBA/TPA Have Similar Tumor Microenvironments

**Figure 5:**
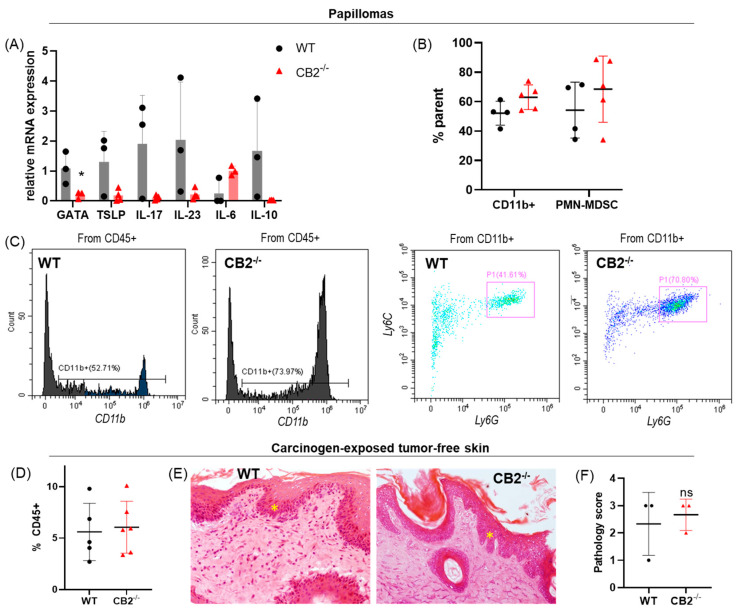
CB2^-/-^ mice exhibit similar tumor microenvironments. (**A**) mRNA expression in papillomas, n ≥ 3 per group. (**B**) Relative frequency of CD45+CD11b+ and PMN-MDSCs (CD11b+Ly6G^hi^Ly6C^int^) in WT and CB2^-/-^ papillomas. (**C**) Representative cytograms of CD11b+ and PMN-MDSC (P1) populations in WT and CB2^-/-^ mice. (**D**) Relative frequency of immune cells (CD45+) in skin exposed to DMBA/TPA treatment that did not have a papilloma visible to the naked eye at the time of harvest (tumor-free). (**E**) Representative histological images of visually tumor-free skin exposed to DMBA/TPA from WT (left) and CB2^-/-^ (right), both with epidermal hyperplasia (yellow star), original magnification 40×. (**F**) Severity of dysplasia according to the same scale as in Figure 1. n ≥ 3 per group, Student’s *t* test, * *p* < 0.05. n = 3 per group, Mann–Whitney *U* test, ns, not significant.

We assessed the mRNA expression of critical players in skin carcinogenesis (IL-6, IL-10, IL-23, IL-17, TSLP, and GATA-3, Table 1) [16,17,39,40]. While GATA-3 appears to be downregulated in the knockout mice (*p* = 0.049), no other significant differences were found, although a trend of reduced TSLP, IL-17, IL-23, and IL-10 expression and increased IL-6 expression were also observed (Figure 5A).

Because CD11b+ cells are known to be upregulated in the bone marrow of CB2^-/-^ mice at steady state or in high inflammatory status [35,41,42], we assessed the papillomas for myeloid cells. Flow cytometry analysis of papillomas showed generally low immune cell infiltration in both genotypes; however, CD11b+ myeloid cells and pro-tumor PMN-MDSCs trended higher in the knockout mice, which was corroborated by higher IL-6 (produced by myeloid cells) mRNA expression in CB2^-/-^ papillomas (Figure 5A–C). Immune cell infiltration into tumor-free carcinogen-exposed skin (no visible papilloma) was also minimal in both groups—averaging five percent (Figure 5D), a comparable level to healthy, naïve skin in mice of this background [43]. Histological analysis of carcinogen-exposed tumor-free skin also showed a low inflammatory infiltrate (Figure 5D,E) and showed no differences in dysplastic severity(Figure 5E,F).

### 2.6. CB2 Expression Is Upregulated in Carcinogen-Exposed Skin of WT Mice

**Figure 6:**
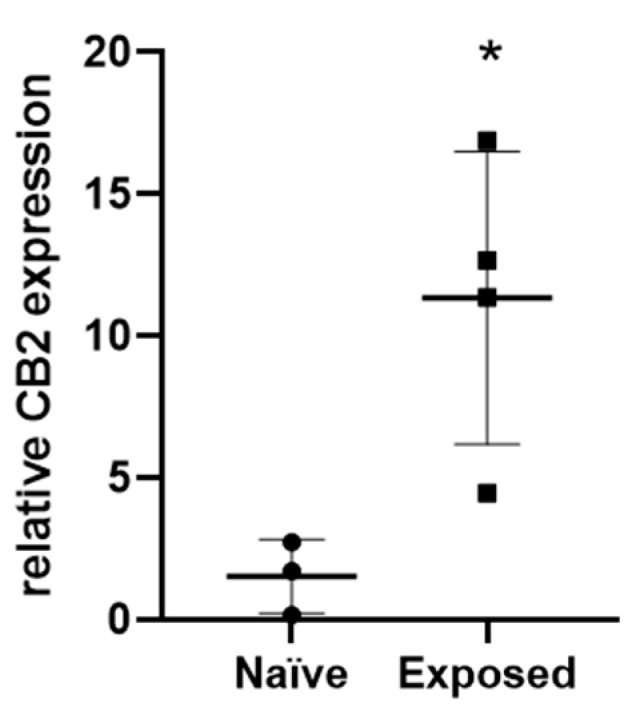
CB2 expression is upregulated in carcinogen-exposed tumor-free skin (‘Exposed’) compared to naïve (ventral) skin in WT mice. Relative mRNA expression of CB2 in naïve compared to exposed skin. n ≥ 3 per group. Student’s *t*-test, * *p* < 0.05.

Because minimal inflammatory infiltrate was seen in the papillomas and carcinogen-exposed skin, coupled with evidence from case studies that cannabinoid receptor activation in non-immunological cells plays a role in cancer regression, we hypothesized a role of CB2 on keratinocytes. Therefore, we measured CB2 expression and found that it is eight-fold higher in carcinogen-exposed skin compared to naïve skin in WT mice (*p* = 0.026, Figure 6).

### 2.7. CB2^-/-^ Mice Treated with DMBA/TPA Have an Altered Systemic Immune Profile

**Figure 7:**
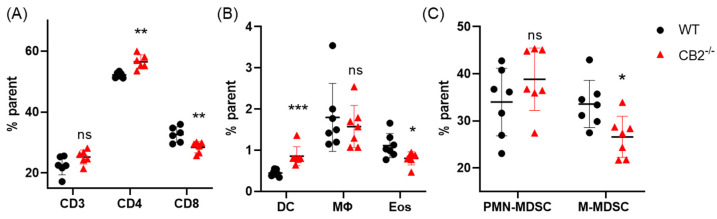
CB2 receptor alters balance between pro-tumor and anti-tumor cells in the spleen in mice receiving DMBA/TPA. (**A**) Relative frequency of CD3+, CD3+CD4+, and CD3+CD8+ T cells in the spleen. (**B**) Relative frequency of CD11b^hi^ Cd11c^hi^ (DC), CD11b+F480+ (macrophage, MΦ), and CD11b+SiglecF+ (eosinophil, Eos). (**C**) Relative frequency of CD11b+Ly6G^hi^Ly6C^int^ (PMN-MDSC) and CD11b+Ly6G-Ly6C^hi^ (M-MDSC). All values expressed as percent of parent population as determined by flow cytometry. WT, n ≥ 6; CB2^-/-^, n ≥ 6. Student’s *t*-test, * *p* < 0.05, ** *p* < 0.01, *** *p* < 0.001, ns, not significant.

Previous reports have shown that peripheral CD4+ and CD8+ T cells play antagonistic roles in skin carcinogenesis, wherein CD8+ T cells are protective against tumor development and CD4+ cells are pro-tumorigenic [44,45]. Because CB2 is expressed on T cells, we hypothesized that T cells play a role in the anti-tumorigenicity of endogenous CB2 activation [46]. Indeed, using flow cytometry, we found that the ratio of CD4:CD8 T cells in the spleen was significantly increased, while the overall number of T cells (CD3+) remained the same, ~24% for both genotypes (Figure 7A). CD8+ T cells were significantly decreased in the spleens of CB2^-/-^ mice (*p* = 0.008), while CD4+ T cells were significantly higher (*p* = 0.002, Figure 7A). Additionally, there have been reports showing that under high inflammatory conditions, such as sepsis and multiple sclerosis, the myeloid compartment of CB2^-/-^ mice is dysregulated [41,42]. Interestingly, we found that in the CB2^-/-^ mice, while macrophages and PMN-MDSCs showed no differences between groups, anti-tumor eosinophils and M-MDSCs were significantly decreased (*p* = 0.031 and *p* = 0.02, respectively), and DCs were increased two-fold (*p* = 0.0008, Figure 7B,C).

## 3. Discussion

We show here that mice lacking the CB2 receptor have an increased risk for spontaneous skin lesions, and in a chemical model of skin carcinogenesis, have heightened papilloma development, less tumor regression, and an altered immune profile in the spleen. Remarkably, the CB2^-/-^ mice do not exhibit the resistance to DMBA/TPA treatment typically seen in C57Bl/6J mice. C57BL/6J mice are considerably resistant to the multi-stage chemical carcinogenesis model using DMBA/TPA, wherein around 40% of mice will develop papillomas and the conversion frequency to carcinoma is less than 1% [6,47]. Rather than using a sensitive mouse strain (i.e., SENCAR, FVB/N), in which the cancer progression is rampant and conversion frequency is high, we chose a resistant strain to isolate the differences in endogenous CB2 activation, as the sensitive strains have clear genetic loci contributing to cancer susceptibility [48,49,50]. Although papilloma development was observed in all mice, the CB2^-/-^ mice were much less resistant to the development of persistent and/or larger papillomas and they exhibited much less spontaneous regression compared to the WT mice.

Previous reports showed that exogenous cannabinoids could induce tumor regression [51,52]; one study also demonstrated the anti-tumor activity of CB2 agonists on epidermal cells and that anandamide (a weak CB2 agonist) selectively induces cell death in tumorigenic keratinocytes [53]. However, to our knowledge, this work is the first evidence of endogenous CB2 activation as a promoter of spontaneous regression.

The progression versus regression pattern was observed in both genotypes and explains the variation in qPCR and flow cytometry data, as at the time the specific papillomas were collected and analyzed, some were regressing and others proliferating. Human clinical data has shown that increased CD4+ T cell infiltration is enhanced in spontaneously regressing NMSC, and in mice, the depletion of CD4+ T cells enhances susceptibility to radiation-induced skin tumors [45,54,55]. Similarly, our data indicates that CB2 expression plays a role in papilloma development via the modulation of the immune cell profile. Additionally, CD4+ T cell markers (IL-17 and IL-10) are higher in the WT mice and GATA-3—whose deficiency leads to impaired CD4+ T cell survival—trends lower in CB2^-/-^ mice. While differences were not statistically significant due to the likely heterogeneity of progressing versus regressing tumors, it is interesting to note that in contrast to the WT controls, none of the CB2^-/-^ specimens had a high expression of the CD4+ T cell markers and genes involved in tumor regression (Figure 5A), suggesting a mechanism for CB2 contribution to tumor regression [16,56]

Although splenic T cell and myeloid cell populations were significantly altered in the knockout mice, the same pattern was not necessarily observed in the papillomas themselves, although a trend towards upregulated PMN-MDSCs and altered expression of inflammatory markers was seen. Overall, minimal differences were observed at the cellular and molecular levels in the carcinogen-exposed tumor-free skin between the two genotypes, while macroscopically the differences in papilloma formation and size were profound. Minimal differences in the carcinogen-exposed tumor-free skin were most likely observed due to varying stages of papilloma development at the microscopic level.

It is important to note that TSLP secreted by the tumor activates myeloid DCs; however, in the skin, specifically in keratinocytes, TSLP exhibits CD4+ T cell-mediated anti-tumor activity [13]. In this study, systemic myeloid DCs (CD11b+CD11c+) were significantly upregulated along with CD4+ T cells in the CB2^-/-^ mice. It is possible that there is a shift in these mice towards the Th2 phenotype, which facilitates tumor growth [57]. Notably, TSLP mRNA expression was not significantly different in the papillomas between groups, although it trended lower in the knockout mice and remained at the limit of detectability in all the CB2^-/-^ mice. Very little immune cell infiltration was seen in the papillomas of either group in the histological analysis. It has previously been shown that DCs of CB2^-/-^ mice have altered maturation phenotypes, and that the immune responses mediated by these cells are reduced [58]. The reason for such a dichotomy, i.e., altered immune profile in the spleen of CB2^-/-^ mice, with no differences in the papillomas, remains elusive. It is possible that the mechanism by which heightened papillomas in mice lacking the CB2 receptor occurs is mediated by the decreased expression of TSLP at the site of the tumor, and while DCs are upregulated systemically, they are not recruited to the affected tissue. Another probable explanation is that the CB2-deficient DCs shift naïve CD4+ T cells towards a Th2 subtype, thus preventing tumor cell apoptosis. Additionally, CB2 agonists may suppress the immune response by inducing apoptosis in DCs, mediated by NF-κB, which could also explain the apparent increase in splenic DCs in the knockout [59].

The increase in CB2 expression in the carcinogen-exposed skin compared to naïve skin shows the possibility that the lack of expression of CB2 in the keratinocytes, rather than in the immune cells, plays a role in heightened papilloma development. In skin carcinogenesis models, hyper-proliferation of keratinocytes is induced, which has a close relationship with pro-inflammatory cytokine secretion [60]. It has previously been shown that endocannabinoids inhibit the proliferation of keratinocytes and elevated expression of CB2 in these cells may prevent tumor growth [53,61,62,63]. This would explain why only CB2^-/-^ mice had large tumors with virtually none in the WT mice.

When comparing the expression and activation levels of various proangiogenic factors in untreated and cannabinoid-treated skin cancers, these factors were significantly reduced in cannabinoid-treated tumors compared to vehicle-treated tumors, supporting the idea that the anti-tumorigenic actions of CB2 are mediated at least in part by the activation of CB2 signaling in skin tumor cells [52,64]. Our data reflects this latter notion, as we found minimal differences in the immune cells within the tumor microenvironment of WT and CB2^-/-^ mice receiving DMBA/TPA coupled with significant systemic immune alterations and significant differences in papilloma development, showing that endogenous CB2 activation is potentially altering the activity and expression of specific tumor growth factors.

It has been shown previously that in humans, CB2 expression is upregulated in cancer and is positively associated with cancer severity, including squamous cell carcinoma found on the skin [52]. Corroboratively, we have shown that CB2 is upregulated in the papillomas of DMBA/TPA-treated WT mice compared to naïve skin from the same mice. While a positive correlation with cancer severity does not suffice to determine whether CB2 activation is beneficial or deleterious to this condition, our findings indicate that CB2 expression protects against skin cancer progression in this model of NMSC. The elevated CB2 expression might therefore contribute to restraining tumor growth via systemic effects on immune cells and/or local actions in keratinocytes. If translated to humans, CB2 activation via selective agonists has a clear therapeutic potential for the treatment of NMSC. We show that endogenous CB2 activation lowers the risk for spontaneous cancer development in aging mice and papilloma development in a chemically-induced model of skin carcinogenesis. CB2 activation can modulate the systemic immune response and reduce tumorigenesis, either by an immune-mediated response involving the alteration of T cells and myeloid cells, or by the modulation of keratinocyte proliferation. This implies that CB2 could have an anti-tumorigenic role in skin cancer and serve as a potential treatment target.

## 4. Materials and Methods

### 4.1. Skin Cancer Induction

Tumor initiation was accomplished by a single application of 25 µg (97.5 nmol) of 7,12-dimethylbenz[a]anthracene (Sigma-Aldrich, St. Louis, MO, USA) diluted in 100 µL of acetone to the backs of 6–7 week-old male C57BlJ/6 WT and CB2^-/-^ mice two days after shaving [6]. After one week, 4 µg (6.5 nmol) of the tumor promoting agent 12-O-tetradecanoylphorbol-13-acetate (Sigma-Aldrich, St. Louis, MO, USA) in 40 µL of acetone was applied twice a week for 27 weeks [6]. The number and size of >1mm tumor lesions were recorded weekly.

### 4.2. Flow Cytometry

After removing spleens, single-cell suspensions were obtained by mashing the organs over a 70 μm cell strainer (Fisher Scientific, Hampton, NH, USA). Bone marrow cells were flushed from tibia and filtered through a 70 μm cell strainer. Red blood cells were lysed using ACK lysing buffer (Gibco, New York, NY, USA). T cells were stained with anti-CD3-FITC, anti-CD4-PE, and anti-CD8-APC for 30 min on ice, while myeloid cells were stained with anti-CD45-Pacific Blue, anti-CD11b-PE-Cy7, anti-CD11c-PE, anti-Ly6G-FITC, anti-Ly6C-PerCP-Cy5.5, and anti-Siglec-F-APC or anti-F4/80-APC for 45 min on ice. After staining, cells were washed twice with PBS and the fluorescence was assessed with a CytoFlex5L (Beckman Coulter, Brea, CA, USA). DCs were classified as CD45+CD11b+CD11c+, macrophages as CD45+CD11b+F4/80+, eosinophils as CD45+CD11b+Siglec-F+, PMN-MDSCs as CD45+CD11b+CD11c^lo/neg^Ly6G^hi^Ly6C^int^, and M-MDSCs as CD45+CD11b+CD11c^lo/neg^Ly6G-Ly6C^hi^. B cells were identified using B220-PE and IgM-APC. All antibodies were purchased from BioLegend (San Diego, CA, USA). After processing, cells were read on a CytoFlex LX (Beckman Coulter, Brea, CA, USA), and analyzed using CytExpert^®^ (Beckman Coulter, Brea, CA, USA).

### 4.3. Histology

Mice skin was harvested and fixed in 4% paraformaldehyde. Specimens were then paraffin-embedded, sliced into 5 µm sections, and stained with hematoxylin–eosin (H&E), according to Bialkowska et al. [65]. All pathological analyses were performed by a board-certified pathologist. Abnormal tissue or cells were graded based on a modification and simplification of the dysplastic lesions seen in human pre-malignant and malignant lesions: 0–normal tissue; 1-chronic inflammation, ulceration, atrophy, or hyperplasia; 2–cellular atypia, metaplasia, or mitotic figures; 3-dysplasia; 4-positive carcinoma [34,66]. Cancer severity as well as the occurrence of pre-cancerous and cancerous lesions in the CB2^-/-^ mice were compared to the situation in the WT.

### 4.4. RNA Extraction, cDNA Synthesis, and Quantitative Real-Time Polymerase Chain Reaction (qPCR)

Total RNA was extracted from the papillomas of similar size of each mouse using TRIzol reagent (Invitrogen, Carlsbad, CA, USA), and qPCR was performed using cDNA generated from 1 µg of total RNA with a cDNA synthesis kit (Quantabio, Beverly, MA, USA). qPCR reactions were carried out on 20 ng cDNA per reaction using SYBR Green PCR master mix (Quantabio, Beverly, MA, USA) using a Step-One (Thermo Fisher, Waltham, MA, USA) analysis system. Relative expression values were quantitated using the comparative cycle threshold method and normalized to mouse β-actin. For the evaluation of CB2 expression in ventral skin (non-exposed) compared to exposed skin, β-2-microglobulin (β2M) was used as the housekeeping gene. The following primers were used in Table 1.

### 4.5. Genotyping 

Genomic DNA was extracted from tail clippings and Extracta DNA Prep for PCR (Quantabio, Beverly, MA, USA). PCR was performed with DreamTaq Green PCR master mix (Thermo Scientific, Waltham, MA, USA). The following primers were used: CB2^-/-^: 5′-AGCGCATGCTCCAGACTGCCT-3′ AGCGCATGCTCCAGACTGCCT, CB2^+/+^ 5′-GTGCTGGGCAGCAGAGCGAATC-3′, and CB2 common antisense: 5′-GTCGACTCCAACGCTATCTTC-3′.

### 4.6. Statistical Analysis

All analyses were conducted using GraphPad Prism v9.0. Data were analyzed by Student’s *t*-test or Mann–Whitney U test for continuous variables. Differences in tumor count between groups for the duration of the experiment were determined using two-way ANOVA for repeated measures over time. All results are expressed as mean values ±SD unless otherwise indicated. *p* < 0.05 was considered statistically significant.

## Figures and Tables

**Table 1:** Real-time PCR primer sequences.

	Primer sequence 5′-3′
GATA3_F	GTGGTCACACTCGGATTCCT
GATA3_R	GCAAAAAGGAGGGTTTAGGG
TSLP_F	AGGCTACCCTGAAACTGAG
TSLP_R	GGAGATTGCATGAAGGAATACC
IL-17_F	ACCGCAATGAAGACCCTGAT
IL-17_R	TCCCTCCGCATTGACACA
IL-23_F	GACAACAGCCAGTTCTGCTT
IL-23_R	AGGGAGGTGTGAAGTTGCTC
IL-10_F	TGAGGCGCTGTCGTCATCGATTTCTCCC
IL-10_R	ACCTGCTCCACTGCCTTGCT
IL-6_F	CCGGAGAGGAGACTTCACAG
IL-6_R	GGAAATTGGGGTAGGAAGGA
β-actin_F	GTCACCCACACTGTGCCCATC
β-actin_R	CCGTCAGGCAGCTCATAGCTC
β2M_F	TGCTACTCGGCGCTTCAGTC
β2M_R	AGGCGGGTGGAACTGTGTTAC
CB2_F	TGACCATGACCTTCACAGCC
CB2_R	GGTAGGCGGGTAACACAGAC

## Data Availability

Data were generated by the authors and are available upon request. Genomic study data, sources, and software used are included in the article and/or Supporting Information.

## Supplementary Materials

The following supporting information can be downloaded at: https://www.mdpi.com/article/10.3390/ijms24097773/s1.
