# Supplementary material for: The Anti-Tumorigenic Role of Cannabinoid Receptor 2 in Non-Melanoma Skin Cancer
Source: Int J Mol Sci. 2023 Apr 24;24(9):7773. doi: 10.3390/ijms24097773 (PMC10178456; doi:10.3390/ijms24097773)
Supplement: Supplementary file 1 [file ijms-24-07773-s001.zip › ijms-2353733-supplementary.pdf]

Table S1. Spontaneous regression and new papilloma formation per mouse in WT and CB2$^{-/-}$ mice receiving DMBA/TPA cancer induction.

Week		WT							CB2-/-						
0.00		0.00	0.00	0.00	0.00	0.00	0.00	0.00	0.00	0.00	0.00	0.00	0.00	0.00	0.00
17.00		1.00	2.00	1.00	0.00	1.00	1.00	1.00	3.00	2.00	2.00	4.00	5.00	0.00	0.00
	# of SR events	0.00	0.00	0.00	0.00	0.00	0.00	0.00	0.00	0.00	0.00	0.00	0.00	0.00	0.00
	new papilloma	1.00	2.00	1.00	0.00	1.00	1.00	1.00	3.00	2.00	2.00	4.00	5.00	0.00	0.00
	rate of SR	0.00	0.00	0.00	0.00	0.00	0.00	0.00	0.00	0.00	0.00	0.00	0.00	0.00	0.00
18.00		0.00	1.00	0.00	0.00	3.00	0.00	0.00	1.00	2.00	4.00	4.00	1.00	2.00	4.00
	# of SR events	1.00	1.00	1.00	0.00	0.00	1.00	1.00	2.00	0.00	1.00	0.00	4.00	0.00	0.00
	new papilloma	0.00	0.00	0.00	0.00	2.00	0.00	0.00	0.00	0.00	3.00	0.00	0.00	2.00	4.00
	rate of SR	100.00	50.00	100.00	N/A	0.00	100.00	100.00	66.67	0.00	0.00	0.00	80.00	N/A	N/A
19.00		1.00	0.00	0.00	0.00	2.00	1.00	1.00	3.00	2.00	6.00	6.00	1.00	1.00	4.00
	# of SR events	0.00	1.00	0.00	0.00	1.00	0.00	0.00	0.00	0.00	0.00	1.00	0.00	1.00	1.00
	new papilloma	1.00	0.00	0.00	0.00	0.00	1.00	1.00	2.00	0.00	2.00	3.00	0.00	0.00	1.00
	rate of SR	N/A	100.00	N/A	N/A	33.33	N/A	N/A	0.00	0.00	0.00	0.00	0.00	50.00	25.00
20.00		0.00	2.00	0.00	1.00	2.00	0.00	3.00	5.00	4.00	8.00	4.00	0.00	1.00	1.00
	# of SR events	1.00	0.00	0.00	0.00	0.00	1.00	0.00	0.00	0.00	0.00	2.00	1.00	0.00	3.00
	new papilloma	0.00	2.00	0.00	1.00	0.00	0.00	2.00	2.00	2.00	2.00	0.00	0.00	0.00	0.00
	rate of SR	100.00	N/A	N/A	N/A	0.00	100.00	0.00	0.00	0.00	0.00	33.33	100.00	0.00	75.00
21.00		4.00	2.00	1.00	1.00	2.00	0.00	0.00	5.00	4.00	8.00	6.00	2.00	2.00	1.00
	# of SR events	0.00	1.00	0.00	0.00	1.00	0.00	3.00	0.00	0.00	1.00	0.00	0.00	0.00	0.00
	new papilloma	4.00	1.00	1.00	0.00	1.00	0.00	0.00	0.00	0.00	1.00	2.00	2.00	1.00	0.00
	rate of SR	N/A	50.00	N/A	0.00	50.00	N/A	100.00	0.00	0.00	12.50	0.00	N/A	0.00	0.00
22.00		1.00	2.00	1.00	1.00	3.00	0.00	0.00	5.00	4.00	11.00	6.00	4.00	2.00	8.00
	# of SR events	4.00	2.00	0.00	0.00	1.00	0.00	0.00	2.00	1.00	1.00	0.00	2.00	0.00	0.00
	new papilloma	1.00	0.00	0.00	0.00	2.00	0.00	0.00	7.00	1.00	4.00	0.00	4.00	0.00	7.00
	rate of SR	100.00	100.00	0.00	0.00	50.00	N/A	N/A	40.00	25.00	12.50	0.00	100.00	0.00	0.00
23.00		4.00	3.00	2.00	4.00	6.00	5.00	3.00	9.00	5.00	13.00	7.00	5.00	8.00	11.00
	# of SR events	1.00	2.00	1.00	1.00	1.00	0.00	2.00	2.00	0.00	0.00	0.00	0.00	1.00	1.00
	new papilloma	4.00	3.00	2.00	4.00	4.00	5.00	5.00	6.00	1.00	2.00	1.00	1.00	7.00	4.00
	rate of SR	25.00	100.00	N/A	N/A	100.00			100.00	0.00	0.00	N/A	0.00	N/A	N/A
24.00		1.00	2.00	3.00	3.00	4.00	2.00	1.00	7.00	5.00	11.00	8.00	3.00	4.00	8.00
	# of SR events	3.00	1.00	2.00	1.00	2.00	5.00	3.00	2.00	0.00	2.00	1.00	2.00	4.00	3.00
	new papilloma	0.00	0.00	3.00	0.00	0.00	2.00	1.00	0.00	0.00	0.00	2.00	0.00	0.00	0.00
	rate of SR	75.00	33.33	100.00	25.00	33.33	100.00	100.00	22.22	0.00	15.38	14.29	40.00	50.00	27.27
25.00		2.00	7.00	2.00	3.00	7.00	8.00	3.00	9.00	6.00	13.00	13.00	7.00	9.00	15.00
	# of SR events	0.00	2.00	3.00	0.00	0.00	2.00	1.00	1.00	0.00	0.00	0.00	0.00	0.00	1.00
	new papilloma	1.00	7.00	2.00	0.00	3.00	8.00	3.00	3.00	1.00	2.00	5.00	4.00	5.00	8.00
	rate of SR	0.00	100.00	100.00	0.00	0.00	100.00	100.00	14.29	0.00	0.00	0.00	0.00	0.00	12.50
26.00		2.00	7.00	2.00	3.00	3.00	4.00	0.00	10.00	5.00	15.00	8.00	5.00	5.00	14.00
	# of SR events	2.00	6.00	1.00	1.00	4.00	5.00	3.00	1.00	1.00	0.00	5.00	2.00	4.00	1.00
	new papilloma	2.00	6.00	1.00	1.00	0.00	1.00	0.00	2.00	0.00	2.00	0.00	0.00	0.00	0.00
	rate of SR	100.00	85.71	50.00	33.33	57.14	62.50	100.00	11.11	16.67	0.00	38.46	28.57	44.44	6.67
26.50		2.00	4.00	3.00	4.00	7.00	6.00	4.00	14.00	6.00	16.00	12.00	5.00	8.00	12.00
	# of SR events	2.00	3.00	2.00	0.00	2.00	2.00	0.00	0.00	0.00	0.00	1.00	0.00	0.00	2.00
	new papilloma	2.00	0.00	3.00	1.00	6.00	4.00	4.00	4.00	1.00	1.00	5.00	0.00	3.00	0.00
	rate of SR	100.00	42.86	100.00	0.00	66.67	50.00	N/A	0.00	0.00	0.00	12.50	0.00	0.00	14.29
27.40		2.00	3.00	0.00	4.00	4.00	6.00	1.00	9.00	8.00	21.00	8.00	6.00	5.00	14.00
	# of SR events	1.00	2.00	3.00	1.00	4.00	1.00	3.00	5.00	0.00	0.00	4.00	0.00	3.00	0.00
	new papilloma	1.00	1.00	0.00	1.00	1.00	1.00	0.00	0.00	2.00	5.00	0.00	1.00	0.00	2.00
	rate of SR	50.00	50.00	100.00	25.00	57.14	16.67	75.00	35.71	0.00	0.00	33.33	0.00	37.50	0.00

SR, spontaneous regression; rate of SR- percent of regressing papillomas
